# Association between *COMT* gene rs165599 SNP and schizophrenia: A meta‐analysis of case‐control studies

**DOI:** 10.1002/mgg3.468

**Published:** 2018-08-30

**Authors:** Harika Gozde Gozukara Bag

**Affiliations:** ^1^ Faculty of Medicine, Department of Biostatistics & Medical Informatics Inonu University Malatya Turkey

**Keywords:** *COMT*, meta‐analysis, odds ratio, schizophrenia, SNP

## Abstract

**Background:**

There are many studies with different results that examine the association between Catechol‐*O*‐MethylTransferase (*COMT*) gene single‐nucleotide polymorphisms (SNPs) and schizophrenia. In this study, the aim was to conduct a meta‐analysis to achieve a pooled effect size of the association between *COMT* gene rs165599 SNP and schizophrenia.

**Methods:**

Odds ratio (OR) was used as an effect size to determine the association between schizophrenia and the SNP. The pooled ORs were achieved under four different genetic models. When the heterogeneity among studies was high the DerSimonian‐Laird random‐effects model, otherwise the Mantel‐Haenszel fixed‐effects model was used. Publication bias was evaluated by Egger's test.

**Results:**

Under different genetic models no statistically significant association was found between rs165599 SNP and schizophrenia by meta‐analyses consist of 20 independent studies. There was high heterogeneity among studies, for the possible reason the population differences, although the subgroup analyzes reduced the heterogeneity, no association was obtained. However, the sex‐specific estimation of the females showed that to be a G allele carrier is a risk factor for schizophrenia (OR = 1.366 [95% confidence interval = 1.094–1.706]) compared to AA homozygous.

**Conclusion:**

The *COMT* gene rs165599 SNP does not appear to be a single‐risk factor for schizophrenia.

## 1 INTRODUCTION

Schizophrenia (MIM # 181500) is a complex psychiatric disorder in which genetic and environmental factors play a role together. Catechol‐O‐methyl transferase (*COMT* [MIM # 116790])is the key enzyme in the catabolism of neurotransmitter dopamine. The *COMT* gene has been mapped to 22q11.2 (de Chaldée et al., 2001; Grossman, Emanuel, & Budarf, 1992). Both linkage and association studies implied that this locus is related with schizophrenia (Collier & Li, 2003; Karayiorgou & Gogos, 1997). The microdeletions in this region are associated with a number of syndromes including Velocardiofacial syndrome (VCFS), Di George Syndrome (de Chaldée et al., 2001). In addition to these deficits, VCFS patients also exhibit an increased incidence of psychiatric and behavioral disorders, including schizophrenia and the rate of schizophrenia in VCFS patients is reported to be around 24%, whereas the incidence of schizophrenia in the general population is only 1% (Murphy, Jones, & Owen, 1999). Due to its genomic location and its function in the catabolism of dopamine *COMT* is considered as a strong candidate gene for schizophrenia (Acar, Sözen, Gözükara, Orman, & Kartalci, 2015).

Therefore, studies investigating the association between schizophrenia and *COMT* gene are increasing day by day. However, some of these independent studies cause confusion due to their different results. Also, the studies with small sample sizes which obtain negative results may lack the necessary power to detect small effects and also population admixtures could obscure the results (Chen, Wang, O'Neill, Walsh, & Kendler, 2004).

Meta‐analysis is a method of statistically combining the results of multiple independent studies conducted on the same subject. Many meta‐analyses have been performed and updated for the most widely studied rs4680 single‐nucleotide polymorphism (SNP) (Barnett, Jones, Robbins, & Müller, 2007; Costas et al., 2011; Fan et al., 2005; Glatt, Faraone, & Tsuang, 2003; Okochi et al., 2009; Taylor, 2018). Also, another common studied SNP is rs165599 (HGVS Name: c.*522G>A). Only one meta‐analysis was performed for rs165599 SNP included both family‐based and case‐control studies (Okochi et al., 2009). In this study, we aimed to update the pooled effect size estimation by combining the results of studies with appropriate meta‐analytical methods that examine the association between *COMT* gene rs165599 single‐nucleotide polymorphism and schizophrenia just for case‐control studies.

## 2 MATERIAL AND METHODS

### 2.1 Literature search

**Figure 1:**
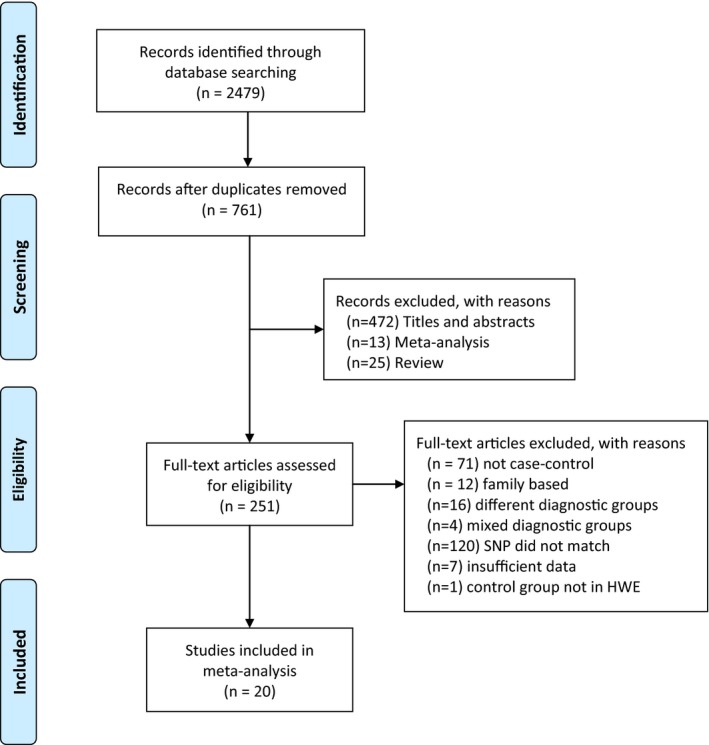
Flow chart of the selection process of the studies included in meta‐analysis

By using the search words “schizophrenia,” “*COMT,*” or “catechol‐*O*‐methyltransferase,” articles were searched on databases (PubMed, Web of Science, Google Scholar) date up to 2017 January. To find other appropriate studies, we also examined the references of the key studies. When there is no detailed information for calculation of the effect size, we tried to contact the authors directly. The selection process of the studies included in the meta‐analysis is given detailed by the flow diagram in Figure 1.

### 2.2 Inclusion and exclusion criteria

The studies which: (a) concern the association between *COMT* SNPs and schizophrenia; (b) use healthy individuals as controls in case‐control studies; (c) has a genotype distribution of control population in Hardy‐Weinberg Equilibrium (HWE) were included in meta‐analysis. The studies; on families (family‐based, trio, siblings, twins), in which patients with different psychiatric diagnosis mixed with schizophrenia, involving only patients with violent tendencies and those involving only patients with alcohol and/or substance abuse were excluded. In all studies, the diagnostic criteria were DSM‐IV (Diagnostic and Statistical Manual of Mental Disorders‐4th edition). The publications not in English could not be considered. The characteristics of the 20 studies included in meta‐analysis resulted from the selection process are given in Table 1.

**Table 1:** Characteristics of the included studies in meta‐analysis

References	Country	Population	Sample size (Case/Control)	Sex Male (Case/Control)	Age (Case/Control) (Mean ± *SD*)
Shifman et al. (2002)	Israel	Ashkenazi Jews	724/4,898	461/3,519	NA/NA
Funke et al. (2005)	US	American (Caucasian)	196/467	NA/225	39.4/39.3
Nicodemus et al. (2007)	Germany	German (Caucasian)	501/627	328/299	NA/NA
Nunokawa et al. (2007)	Japan	Japanese (Asian)	398/440	212/230	40.6 ± 14.7/38 ± 10.4
Yu et al. (2007)	China	Han Chinese (Asian)	241/290	129/160	NA/NA
Martorell et al. (2008)	Spain	Catalan (Caucasian)	584/615	389/321	48.8 ± 17.5/43.1 ± 15.3
Chien et al. (2009)	Taiwan	Taiwanese (Asian)	121/110	71/43	37.5 ± 6.8/31.8 ± 7.6
Gupta et al. (2009)	India	South Indian	396/237	240/151	29.63 ± 8.63/31.21 ± 11.16
Okochi et al. (2009)	Japan	Japanese (Asian)	1,104/1,101	628/504	45.4 ± 15.5/38.1 ± 15.2
Park et al. (2009)	Korea	Korean (Asian)	348/393	176/224	44.0 ± 9.3/54.6 ± 9.3
Chen et al. (2011)	Taiwan	Han Chinese (Asian)	434/442	262/294	37.34 ± 12.63/39.21 ± 12.35
Zhang et al. (2012)	China	Han Chinese (Asian)	768/1,348	360/658	33.5 ± 8.7/31.1 ± 13.2
Wright et al. (2012)	South Africa	Xhosa	235/240	195/190	35.76 ± 11.30/35.83 ± 11.72
Cordeiro et al. (2012)	Brazil	Brazilian	245/829	159/565	NA/NA
Altinyazar et al. (2015)	Turkey	Turkish (Caucasian)	178/365	107/204	36.3 ± 10.6/35.7 ± 11.4
Behbahani et al. (2015)	Iran	Iranian	100/127	67/70	36.9 ± 10.2/37.6 ± 9.6
Higashiyama et al. (2016)	Turkey	Turkish (Caucasian)	96/100	66/47	NA/NA
Higashiyama et al. (2016)	Japan	Japanese (Asian)	502/691	273/343	39.2 ± 13.5/46.1 ± 17.9
Dean and Scarr (2016)	Australia	European and Han Chinese	100/100	58/50	44 ± 2/45 ± 1.9
Matsuzaka et al. (2017)	Brazil	Mixed	210/256	146/158	36.03 ± 10.61/37.12 ± 12.19

### 2.3 Meta‐analysis

Odds ratio (OR) is a risk measure commonly used for retrospective case‐control studies. It is investigated retrospectively to determine how much of the case and control groups are exposed to the risk factor that thought to be (Alpar, 2011). OR can be any non‐negative value. Interpretation of the OR; if OR = 1 exposure to the factor is not a risk, if OR > 1 the factor increases the risk, if OR < 1 the factor decreases the risk on interested outcome (Agresti, 2002).

The odds ratio was used as the effect size to evaluate the association between schizophrenia and allele or genotype distribution. *I* ^2^ and Cochran's *Q* test statistics were used for evaluation of heterogeneity among studies. If the heterogeneity was high (*I* ^2 ^> 50% or *p* < 0.05) the DerSimonian‐Laird (DSL) random effects model; otherwise, the Mantel‐Haenszel (MH) fixed effects model was used to achieve the pooled effect size. Meta‐analyses were conducted under four different genetic models: A versus G, AA versus GG, AA versus G‐allele carriers (AG + GG), and A‐allele carriers (AA + AG) versus GG. The significance of the pooled ORs was determined by a z test and publication bias was assessed by Egger's test. In addition, for the studies providing data by sex, pooled estimations are obtained with six studies and four studies, respectively, for allelic and genotypic distributions under these models. Because of the high heterogeneity among all studies included in the meta‐analyses, population subgroup analyzes were performed by eight studies of Asian origin and five studies of Caucasian origin. In all analyses, the significance level was considered to be 0.05 and all analysis performed by STATA 14.0 software (Stata Corporation, College Station, Texas, USA).

## 3 RESULTS

**Figure 2:**
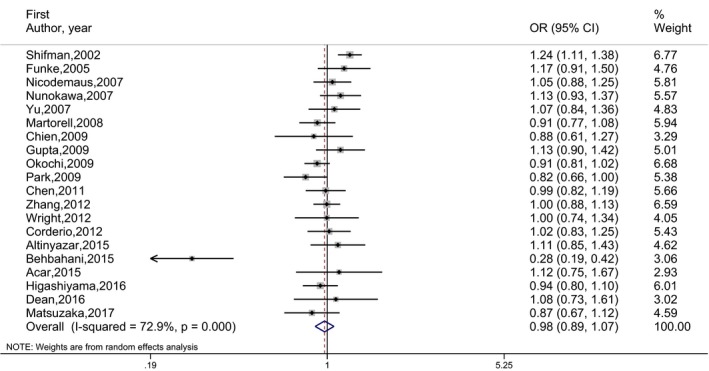
Forest graph for the association between rs165599 SNP and schizophrenia under (A vs. G) genetic model for all studies

**Figure 3:**
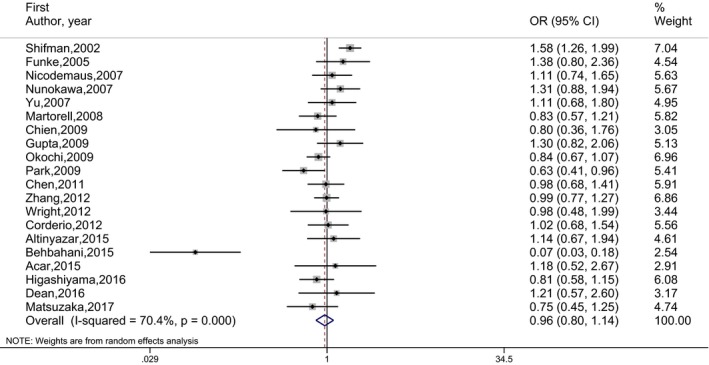
Forest graph for the association between rs165599 SNP and schizophrenia under (AA vs. GG) genetic model for all studies

**Figure 4:**
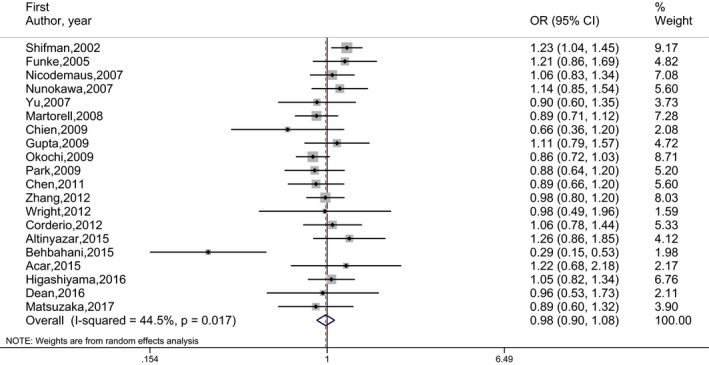
Forest graph for the association between rs165599 SNP and schizophrenia under (AA vs. AG + GG) genetic model for all studies

**Figure 5:**
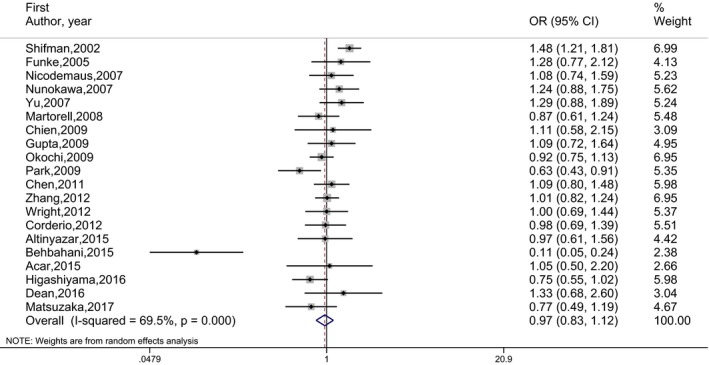
Forest graph for the association between rs165599 SNP and schizophrenia under (AA + AG vs. GG) genetic model for all studies

Literature search resulted in 20 studies that met the inclusion criteria and pooled OR estimates for these studies were performed by appropriate meta‐analytical methods. Common OR estimates and 95% confidence intervals (95% CI) under different genetic models and p values for the *Q* heterogeneity test (*p*_Q_), for the significance of the pooled estimate (*p*_Z_) and for the Egger's bias test (*p*_E_) are given in Table 2. The number of studies included in the meta‐analysis under each genetic model is indicated in parentheses by superscripts in the tables. OR estimates, 95% CI and the weights of 20 independent studies are presented by forest plots in Figures 2, 3, 4, 5. No publication bias was determined by the Egger's test. There was no statistically significant association between schizophrenia and rs165599 SNP according to the results of the meta‐analyses performed under different genetic models.

**Table 2:** The results of the meta‐analyses under different genetic models for all studies

Genetic model	OR (95% CI)	*I* ^2^ (%)	*p* value		
			*p*_Q_	*p*_Z_	*p*_E_
A versus G^[20]^	0.975 (0.893–1.065)	72.9	<0.001	0.576	0.188
AA versus (AG + GG)^[20]^	0.985 (0.896–1.082)	44.5	0.017	0.748	0.178
(AG + AA) versus GG^[20]^	0.967 (0.834–1.121)	69.5	<0.001	0.658	0.144
AA versus GG^[20]^	0.955 (0.802–1.138)	70.4	<0.001	0.607	0.149

Notes

The superscript numbers in brackets represent the number of studies included in meta‐analysis.

*p*_Q_: *p* value for *Q* test; *p*_Z_: *p* value for z test; *p*_E_: *p* value for Egger's bias test.

**Figure 6:**
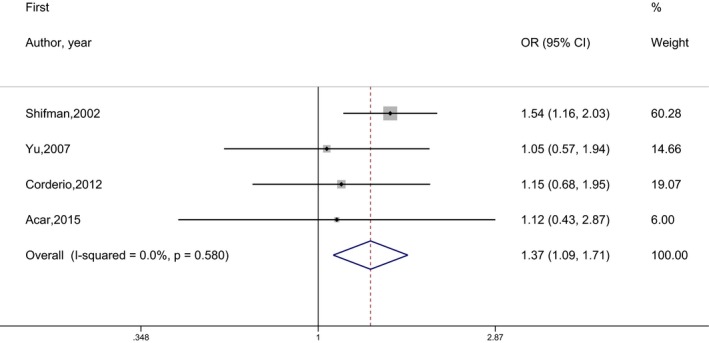
Forest graph for the association between rs165599 SNP and schizophrenia under (AA vs. AG + GG) genetic model for females

Between‐study heterogeneity was observed to be high. Population differences involving ethnicity is thought to be a heterogeneity source. Therefore, eight studies of Asian origin and five studies of Caucasian origin were evaluated separately by meta‐analyses. In the subgroup analyzes, the heterogeneity within populations was remarkably reduced, but no association was found between the disease and the SNP. In addition, in separate analyzes performed with the studies that provided sex‐based data, it was determined that there is no association between schizophrenia and SNP for males, but it is a risk factor to be a G allele carrier for females (Figure 6). The risk of schizophrenia in females with G allele carriers (AG + GG) is found to be 1.366 (95% CI = 1.094–1.706) times higher compared to AA homozygotes. In other words, being an AA homozygous has a protective effect on schizophrenia in females. The results of the meta‐analyses for subgroups are given in Table 3.

**Table 3:** The results of subgroup meta‐analyses under different genetic models

Subgroup	Genetic model	OR (95% CI)	*I* ^2^ (%)	*p* value	
				*p*_Q_	*p*_Z_
Asian	A versus G^[8]^	0.961 (0.904–1.020)	6.3	0.382	0.190
	AA versus (AG + GG)^[8]^	0.937 (0.853–1.029)	0.0	0.635	0.174
	(AG + AA) versus GG^[8]^	0.961 (0.867–1.066)	47.6	0.064	0.452
	AA versus GG^[8]^	0.916 (0.812–1.035)	15.7	0.306	0.158
Caucasian	A versus G^[5]^	1.030 (0.934–1.135)	0.0	0.472	0.559
	AA versus (AG + GG)^[5]^	1.049 (0.918–1.199)	0.0	0.446	0.481
	(AG + AA) versus GG^[5]^	1.014 (0.830–1.239)	0.0	0.799	0.891
	AA/GG^[5]^	1.052 (0.849–1.304)	0.0	0.607	0.641
Female	A versus G^[6]^	1.024 (0.774–1.356)	79.1	<0.001	0.866
	AA versus (AG + GG)^[4]^	**1.366 (1.094**–**1.706)**	0.0	0.580	0.006*
	(AG + AA) versus GG^[4]^	1.359 (0.823–2 243)	63.6	0.041	0.230
	AA versus GG^[4]^	1.470 (0.843–2.563)	59.0	0.063	0.175
Male	A versus G^[6]^	0.679 (0.207–2.225)	99.3	<0.001	0.522
	AA versus (AG + GG)^[4]^	0.492 (0.070–3.442)	99.0	<0.001	0.475
	(AG + AA) versus GG^[4]^	0.639 (0.169–2.418)	97.1	<0.001	0.509
	AA versus GG^[4]^	0.522 (0.084–3.243)	98.0	<0.001	0.485

The superscript numbers in brackets represent the number of studies included in meta‐analysis.

*p*_Q_: *p* value for *Q* test; *p*_Z_: *p* value for z test. *p<0.05.

## 4 DISCUSSION

Many studies have examined the association between polymorphisms in *COMT* gene and schizophrenia. However, there was not a complete consistency in the results of these studies. The aim of this study was to conduct a meta‐analysis for rs165599, one of the suspicious SNPs for schizophrenia. Okochi et al. (2009) conducted a meta‐analysis that considered the case‐control and family studies together. According to their results on 11 studies, there was no association between disease and SNP. In this study, 20 case‐control studies with patients only if diagnosed with schizophrenia were included in the meta‐analysis.

A remarkable study in the literature that examined the association between schizophrenia and *COMT* SNPs was performed by Shifman et al. (2002) with Ashkenazi Jews. According to their findings, rs165599 SNP showed the greatest association with schizophrenia and the G allele is a risk factor. Also, an association with schizophrenia was detected in haplotype analysis of 2 and 3 SNPs for rs737865‐rs4680‐rs165599 SNPs (Shifman et al., 2002). Chien, Liu, Fann, Liu, and Hwu (2009) were found no difference between patients and controls for rs165599 SNP and for the haplotype block in the study of Shifman et al. for Taiwanese. In the study of Altinyazar, Gunderici, Tinaz, and Kirci (2015), haplotypes with 2 and 3 SNPs were studied for rs2075507‐rs4680‐rs165599 SNPs and no association with disease was detected.

Another study, that found an association between schizophrenia and rs165599 SNP, was conducted on the Iranians by Behbahani, Kazemi‐Nezhad, Foroughmand, and Ahmadi (2015). However, as an opposite result to Shifman et al.’s, it was reported that AA and AG increased the risk of schizophrenia when GG genotype was taken as a reference.

Funke et al. (2005), found an association between rs165599 SNP and psychiatric disorders including schizophrenia, schizoaffective disorder, bipolar disorder and major depressive disorder. According to their study having a G allele was reported to increase the risk of psychiatric disorders, while no association between rs165599 SNP and only schizophrenia was determined. In addition, in the haplotype analyzes with four SNPs by adding 278A/G SNP to the haplotype block of Shifman et al.’s, statistically significant results were obtained for all the included psychiatric disorders (Funke et al., 2005). Gupta et al. (2009), in their study of the Indians, reported an association with disease in a different sequence of haplotype block studied by Shifman et al. (2002) and also in the haplotype block with seven SNPs in which other SNPs were also included. In the study of Matsuzaka, Christofolini, and Ota (2017), it is stated that by haplotype analysis of rs4680‐rs165599, an association between G‐A haplotype and schizophrenia was obtained when compared with G‐G haplotype.

Chen, Lu, Yeh, Shih, and Huang (2011) investigated 14 polymorphisms in the *COMT* gene, including rs165599, and found no difference between patients and controls in terms of allele or genotype distributions. In addition, no association was found between the disease and the rs174697‐rs165599‐rs165728 haplotype and by the logistic regression analysis for each SNP in which age and sex were included in the model (Chen et al., 2011). Okochi et al. (2009) besides the meta‐analysis, conducted a case‐control study on Japanese and found no difference between patients and controls in terms of allele and genotype distribution of rs165599 SNP. Also, there was no difference in terms of 2, 3 and 4 SNP haplotypes of seven polymorphisms they studied. In the study of Wright et al. (2012), 14 SNPs were studied in the *COMT* gene, and the association between disease and rs737865 and rs2020917 polymorphisms was found to be significant. Zhang et al. (2012) found no association between schizophrenia and 9 SNPs studied in the *COMT* gene.

When the polymorphism considered in this study was examined according to sex, Yu, Zhang, Huang, Ding, and Li (2007), Martorell et al. (2008), Cordeiro, Silva, and Vallada (2012) and Acar et al. (2015) reported no difference between patients and controls. Chen et al. (2011) stated that there was no difference in analysis made for females and males separately (data not shown). In Ashkenazi Jews, it is reported that there is no association between disease and SNP in males, whereas in females in terms of allele, having G allele and in terms of genotype, being GG are associated with schizophrenia (Shifman et al., 2002). They also reported that the difference between allele and genotype distributions of males and females in the control group was statistically significant. In our study, when the data of the other studies were analyzed, there was no statistically significant difference between the males and females in the control groups due to these distributions. However, in the study of Park et al. (2009), in which the allele distribution by sex is given, whereas no difference was found for males, it has been identified as a risk factor of having A allele for females. The reason for the different identified risky allele in these two studies might be the difference of the ethnic origins of the study groups. In our study, as a result of the meta‐analyses conducted separately by sex, it was concluded that while there is no association for males, having a G allele is a risk factor for females according to pooled estimation. In fact, this result is obtained by the influence of the Shifman et al.’s study because of its high study weight compared to other studies (Figure 6). So, it may be just specific to Ashkenazi Jews. In order to make a more accurate interpretation, more sex‐based investigations need to be done.

In the meta‐analyses including all studies, a high heterogeneity was observed between the ORs of the studies. Therefore, subgroup analyzes were conducted to take the population differences involving ethnicity into account, which is considered to be a possible source of heterogeneity. Populations according to the ethnicity indicated in the studies are pooled as Asians and Caucasians. Heterogeneity among the studies of Asians is considerably reduced and the studies of Caucasians have provided homogeneity. According to within‐population assessments, no association between schizophrenia and SNP was detected under four genetic models (Table 3).

Another source of heterogeneity among studies is thought to be the differences in the selection of control groups (Chen et al., 2011). In some studies, individuals in the control group are assessed and selected by psychiatrists, whereas in some, the family history, current, and past psychiatric conditions of the individuals are questioned with unstructured forms. The control group of some studies consist of hospital staff or blood bank volunteers. In order to achieve correct results in comparison with the patient group, the control group should be exactly separated in terms of the disease.

## 5 CONCLUSION

In our published previous study, it was stated that there was no association between rs165599 SNP and schizophrenia (Acar et al., 2015). Nevertheless, to make clear the heterogeneous findings of the studies by the pooled estimations via meta‐analyses, we concluded that the rs165599 single‐nucleotide polymorphism does not appear to be a single‐risk factor for schizophrenia. It is stated that different haplotypes may be associated with schizophrenia in different populations instead of single‐nucleotide polymorphism due to differences in linkage disequilibrium (LD) among populations (Mukherjee et al., 2010). We thought haplotype analyzes instead of single‐nucleotide polymorphisms will be more informative for future studies.

## CONFLICTS OF INTEREST

The author reports no conflict of interest.
